# Lipoprotein(a) and Hypertension-Mediated Organ Damage in Patients with Essential Hypertension: Associations with Carotid Plaque and Impaired Nocturnal Blood Pressure Dipping

**DOI:** 10.3390/medicina62081561

**Published:** 2026-08-14

**Authors:** Tolga Kunak, Ayşegül Ülgen Kunak, İbrahim Başarıcı

**Affiliations:** 1 Department of Cardiology, Faculty of Medicine, Akdeniz University, 07070 Antalya, Türkiye; dr.tolgakunak@gmail.com (T.K.); ibasarici@gmail.com (İ.B.); 2 Department of Cardiology, Antalya Training and Research Hospital, University of Health Sciences, 07100 Antalya, Türkiye

**Keywords:** lipoprotein(a), hypertension, carotid plaque, nocturnal blood pressure dipping, ambulatory blood pressure monitoring, carotid atherosclerosis

## Abstract

*Background and Objectives*: Lipoprotein(a) [Lp(a)] is an established cardiovascular risk factor associated with atherosclerosis, vascular inflammation, and endothelial dysfunction. However, data regarding its relationship with hypertension-mediated organ damage and circadian blood pressure abnormalities remain limited. We aimed to investigate the association of elevated Lp(a) levels with carotid atherosclerosis and nocturnal blood pressure dipping patterns in patients with hypertension. *Materials and Methods*: This retrospective cross-sectional study included 80 nondiabetic patients with essential hypertension. Patients were categorized according to serum Lp(a) levels into elevated Lp(a) (≥50 mg/dL, *n* = 20) and lower Lp(a) (<50 mg/dL, *n* = 60) groups. All participants underwent carotid ultrasonography, transthoracic echocardiography, ambulatory blood pressure monitoring, and ophthalmologic evaluation. Multivariable logistic and linear regression analyses were performed to determine independent associations between Lp(a), carotid plaque presence, and nocturnal dipping percentage. *Results*: Patients with elevated Lp(a) levels had a significantly higher prevalence of carotid plaque (55.0% vs. 23.3%, *p* = 0.008) and non-dipper hypertension (75.0% vs. 46.7%, *p* = 0.028) compared with patients with lower Lp(a) levels. Serum Lp(a) concentrations were inversely correlated with nocturnal dipping percentage (r = −0.362, *p* = 0.001). In multivariable logistic regression analysis adjusted for age, sex, LDL cholesterol, and active smoking, elevated Lp(a) remained independently associated with carotid plaque presence (OR 3.24, 95% CI 1.06–9.80, *p* = 0.039). In multiple linear regression analysis adjusted for age, sex, LDL cholesterol, and office systolic blood pressure, higher Lp(a) levels remained independently associated with lower nocturnal dipping percentage (β = −0.294, *p* = 0.008). No significant associations were observed between Lp(a) and carotid intima–media thickness, left ventricular hypertrophy, hypertensive retinopathy, or renal functional parameters. *Conclusions*: Elevated Lp(a) levels were independently associated with carotid plaque presence and impaired nocturnal blood pressure decline in hypertensive patients. These findings suggest that Lp(a) may be more closely linked to focal macrovascular atherosclerosis and abnormal circadian blood pressure regulation rather than diffuse hypertensive structural remodeling.

## 1. Introduction

Hypertension remains one of the leading causes of cardiovascular morbidity and mortality worldwide and is strongly associated with both macrovascular and microvascular target organ damage [1]. Despite advances in antihypertensive treatment and cardiovascular prevention strategies, residual cardiovascular risk persists even among adequately treated patients [2]. Therefore, increasing attention has focused on biomarkers that may improve vascular risk stratification and better reflect subclinical vascular injury.

Lipoprotein(a) [Lp(a)] is a genetically determined cardiovascular risk factor with proatherogenic, proinflammatory, and antifibrinolytic properties [3,4]. Elevated Lp(a) has been implicated in endothelial dysfunction, oxidative stress, vascular inflammation, and plaque formation, thereby contributing to atherosclerotic cardiovascular disease [5]. Current European Society of Cardiology/European Atherosclerosis Society guidelines recommend at least one lifetime measurement of Lp(a) to improve cardiovascular risk assessment [6]. Elevated Lp(a) concentrations have been associated with coronary artery disease, ischemic stroke, calcific aortic valve disease, and carotid atherosclerosis [7]. Recent consensus statements have further emphasized its contribution to residual cardiovascular risk despite optimal low-density lipoprotein cholesterol control [8].

Hypertension-mediated organ damage involves both macrovascular and microvascular alterations. Among vascular imaging markers, carotid plaque is considered a clinically relevant indicator of focal atherosclerotic burden, whereas carotid intima–media thickness (IMT) mainly reflects diffuse arterial remodeling associated with chronic hypertension [9,10]. Previous imaging studies have demonstrated that elevated Lp(a) levels are more strongly associated with carotid plaque burden and plaque progression than with carotid IMT, suggesting a preferential role in focal atherosclerotic processes [11,12].

Circadian blood pressure variability is another important determinant of cardiovascular prognosis. A non-dipper blood pressure pattern is associated with an increased risk of stroke, myocardial infarction, chronic kidney disease, and hypertension-mediated organ damage [13,14]. Hypertensive retinopathy represents another manifestation of hypertension-mediated microvascular injury and may reflect systemic endothelial dysfunction [15]. Since endothelial dysfunction, oxidative stress, and vascular inflammation are common mechanisms underlying both elevated Lp(a) levels and impaired nocturnal blood pressure regulation, Lp(a) may also be associated with abnormal circadian blood pressure patterns.

Despite the established role of Lp(a) in atherosclerotic cardiovascular disease, data regarding its relationship with different manifestations of hypertension-mediated organ damage and circadian blood pressure abnormalities remain limited. Therefore, this exploratory study aimed to investigate the association between elevated Lp(a) levels and carotid atherosclerosis, cardiac structural changes, retinal microvascular findings, and ambulatory blood pressure abnormalities in nondiabetic patients with essential hypertension.

## 2. Materials and Methods

### 2.1. Study Design

This single-center, retrospective, cross-sectional exploratory observational study included consecutive adult nondiabetic patients with essential hypertension who were evaluated at a tertiary cardiology outpatient clinic between January 2022 and December 2024. The study protocol was conducted in accordance with the Declaration of Helsinki and was approved by the Institutional Ethics Committee of Akdeniz University (Approval No. TBAEK-434; 30 April 2026).

Hypertension was defined according to the 2023 European Society of Hypertension guideline recommendations [16].

### 2.2. Inclusion and Exclusion Criteria

Eligible participants were adults (≥18 years) with essential hypertension who underwent comprehensive cardiovascular evaluation, including ambulatory blood pressure monitoring (ABPM), carotid ultrasonography, transthoracic echocardiography, and ophthalmologic examination during the study period.

Patients with diabetes mellitus were excluded to minimize the potential confounding effects of diabetes-related macrovascular and microvascular injury, including carotid atherosclerosis, retinal abnormalities, renal dysfunction, and cardiac remodeling.

Patients with known coronary artery disease, previous myocardial infarction, heart failure, moderate-to-severe valvular heart disease, chronic kidney disease (estimated glomerular filtration rate < 60 mL/min/1.73 m^2^), inflammatory or autoimmune disease, active infection, malignancy, chronic liver disease, secondary hypertension, or use of medications known to significantly alter Lp(a) levels, including nicotinic acid and PCSK9 inhibitors, were excluded. Patients with incomplete ambulatory blood pressure monitoring, carotid ultrasonography, echocardiographic, or ophthalmologic data were also excluded.

### 2.3. Study Groups

Patients were stratified into two groups according to serum Lp(a) concentrations: elevated Lp(a) (≥50 mg/dL, *n* = 20) and lower Lp(a) (<50 mg/dL, *n* = 60). The cutoff value of 50 mg/dL was selected according to current guideline recommendations and the previous literature defining increased cardiovascular risk [6].

### 2.4. Sample Size Considerations

Because this was a retrospective, exploratory, single-center observational study, no formal a priori sample size calculation was performed. Consecutive eligible patients who met the predefined inclusion and exclusion criteria during the study period were included. Therefore, the final sample size was determined by the number of eligible patients available during the study period rather than by a predefined sample size estimation.

To assess the adequacy of the primary analysis, a post hoc power analysis was performed using G*Power software (version 3.1; Heinrich Heine University, Düsseldorf, Germany). Based on the observed difference in carotid plaque prevalence between the study groups (23.3% vs. 55.0%), the achieved statistical power was 72.8% at a two-sided α level of 0.05.

### 2.5. Clinical and Laboratory Assessment

Demographic characteristics, medical history, smoking status, office blood pressure measurements, and current antihypertensive medications were retrieved from the electronic medical records. Information regarding hypertension duration, medication timing, statin use, menopausal status, physical activity, alcohol consumption, sleep quality, and obstructive sleep apnea was not consistently available because of the retrospective study design.

Venous blood samples were obtained after overnight fasting for at least 8 h.

Serum Lp(a) concentrations were measured using an immunoturbidimetric assay on a Roche Cobas analyzer (Roche Diagnostics, Mannheim, Germany) according to the manufacturer’s instructions.

Lipid parameters, creatinine, and other biochemical measurements were analyzed using standard laboratory techniques.

Estimated glomerular filtration rate (eGFR) was calculated using the Cockcroft–Gault formula to identify patients with chronic kidney disease for exclusion from the study.

Twenty-four-hour urinary protein and albumin excretion were also evaluated.

### 2.6. Echocardiographic Assessment

Comprehensive transthoracic echocardiography was performed using commercially available ultrasound systems by experienced cardiologists blinded to laboratory findings, according to current guideline recommendations [17]. Echocardiographic parameters were selected to assess hypertension-mediated cardiac target organ damage, with particular focus on left ventricular remodeling. Left ventricular mass index (LVMI) and left ventricular hypertrophy (LVH) were evaluated because they represent established markers of chronic pressure-related cardiac structural changes and cardiovascular risk in patients with hypertension.

Left ventricular dimensions, interventricular septal thickness, posterior wall thickness, left ventricular ejection fraction, and left ventricular mass were measured. Left ventricular mass index (LVMI) was calculated by indexing left ventricular mass to body surface area. Left ventricular hypertrophy (LVH) was defined according to sex-specific guideline criteria.

### 2.7. Carotid Ultrasonographic Assessment

Bilateral carotid ultrasonographic examinations were performed using high-resolution B-mode ultrasonography by a single experienced operator who was blinded to the clinical and laboratory findings.

The bilateral common carotid arteries, carotid bulbs, and proximal internal carotid arteries were systematically examined in all participants.

Mean and maximum carotid IMT measurements were obtained from the common carotid arteries.

Carotid plaque was defined as a focal structure protruding into the arterial lumen with a thickness ≥1.5 mm or >50% greater than the surrounding intima–media complex, according to the Mannheim consensus criteria [18].

### 2.8. Ambulatory Blood Pressure Monitoring

Twenty-four-hour ambulatory blood pressure monitoring (ABPM) was performed using validated oscillometric devices according to international recommendations [14]. ABPM recordings were performed during the same clinical evaluation period after the outpatient visit. Blood pressure measurements were obtained at 15–30 min intervals during daytime and 30–60 min intervals during nighttime. Daytime was defined as the period between 07:00 and 23:00, whereas nighttime was defined as the period between 23:00 and 07:00. Daytime and nighttime systolic and diastolic blood pressure values were calculated as the mean of all valid measurements obtained during the corresponding periods. Recordings with insufficient valid measurements were excluded from the analysis. The nocturnal systolic blood pressure dipping percentage was calculated as [(Daytime SBP − Nighttime SBP)/Daytime SBP] × 100. Patients with a nocturnal systolic blood pressure reduction < 10% were classified as having a non-dipper blood pressure pattern [14].

### 2.9. Ophthalmologic Assessment

Funduscopic examination was performed by experienced ophthalmologists blinded to clinical and laboratory data. Hypertensive retinopathy findings were assessed according to standard clinical criteria [15].

### 2.10. Statistical Analysis

Statistical analyses were performed using IBM SPSS Statistics software (Version 27, IBM Corp., Armonk, NY, USA). Continuous variables were expressed as mean ± standard deviation (SD) or median (IQR), whereas categorical variables were presented as frequencies and percentages. Normality was assessed using visual and analytical methods. Continuous variables were compared using the independent samples *t*-test or the Mann–Whitney U test, as appropriate. Categorical variables were compared using the chi-square test or Fisher’s exact test.

Pearson correlation analysis was performed to evaluate the relationship between continuous serum Lp(a) levels and nocturnal blood pressure dipping percentage. To address the association between continuous serum Lp(a) concentrations and carotid plaque, univariable logistic regression analysis was additionally performed. Variables entered into the multivariable regression models were selected a priori based on their established clinical relevance and previously reported associations with carotid atherosclerosis and nocturnal blood pressure dipping rather than solely on the basis of univariable statistical significance. Multivariable logistic regression analysis was performed to identify independent predictors of carotid plaque. Multiple linear regression analysis was additionally performed to identify independent predictors of nocturnal blood pressure dipping percentage.

Model calibration for the logistic regression analysis was assessed using the Hosmer–Lemeshow goodness-of-fit test.

A two-sided *p* value < 0.05 was considered statistically significant.

## 3. Results

### 3.1. Baseline Characteristics

A total of 80 hypertensive patients were included in the final analysis and stratified according to serum Lp(a) concentration into a low Lp(a) group (<50 mg/dL, *n* = 60) and a high Lp(a) group (≥50 mg/dL, *n* = 20).

Baseline demographic, clinical, laboratory, and imaging characteristics are presented in Table 1. Patients with elevated Lp(a) levels were significantly older than those with lower Lp(a) levels (57.6 ± 9.3 vs. 53.1 ± 6.9 years, *p* = 0.024). As expected, serum Lp(a) concentrations were markedly higher in the elevated Lp(a) group (90.1 ± 39.3 vs. 20.1 ± 12.4 mg/dL, *p* < 0.001).

No significant between-group differences were observed in body mass index, office blood pressure measurements, lipid profile, renal function, echocardiographic parameters, carotid IMT, carotid cross-sectional area, or left ventricular systolic function (all *p* > 0.05). Likewise, sex distribution, active smoking status, and antihypertensive medication use were comparable between the two groups.

Lp(a), lipoprotein(a); BMI, body mass index; bpm, beats per minute; SBP, systolic blood pressure; DBP, diastolic blood pressure; LDL, low-density lipoprotein; HDL, high-density lipoprotein; eGFR, estimated glomerular filtration rate; LVMI, left ventricular mass index; IMT, intima–media thickness; IVS, interventricular septum; PW, posterior wall.

### 3.2. Target Organ Damage and Ambulatory Blood Pressure Findings

Carotid plaque was significantly more prevalent in patients with elevated Lp(a) levels than in those with lower Lp(a) levels (55.0% vs. 23.3%, *p* = 0.008). Likewise, the prevalence of a non-dipper blood pressure pattern was significantly higher in the elevated Lp(a) group (75.0% vs. 46.7%, *p* = 0.028).

In contrast, no significant differences were observed between groups regarding left ventricular hypertrophy, daily proteinuria, daily albuminuria, or hypertensive retinopathy (all *p* > 0.05) (Table 2).

### 3.3. Correlation Analysis

**Figure 1:**
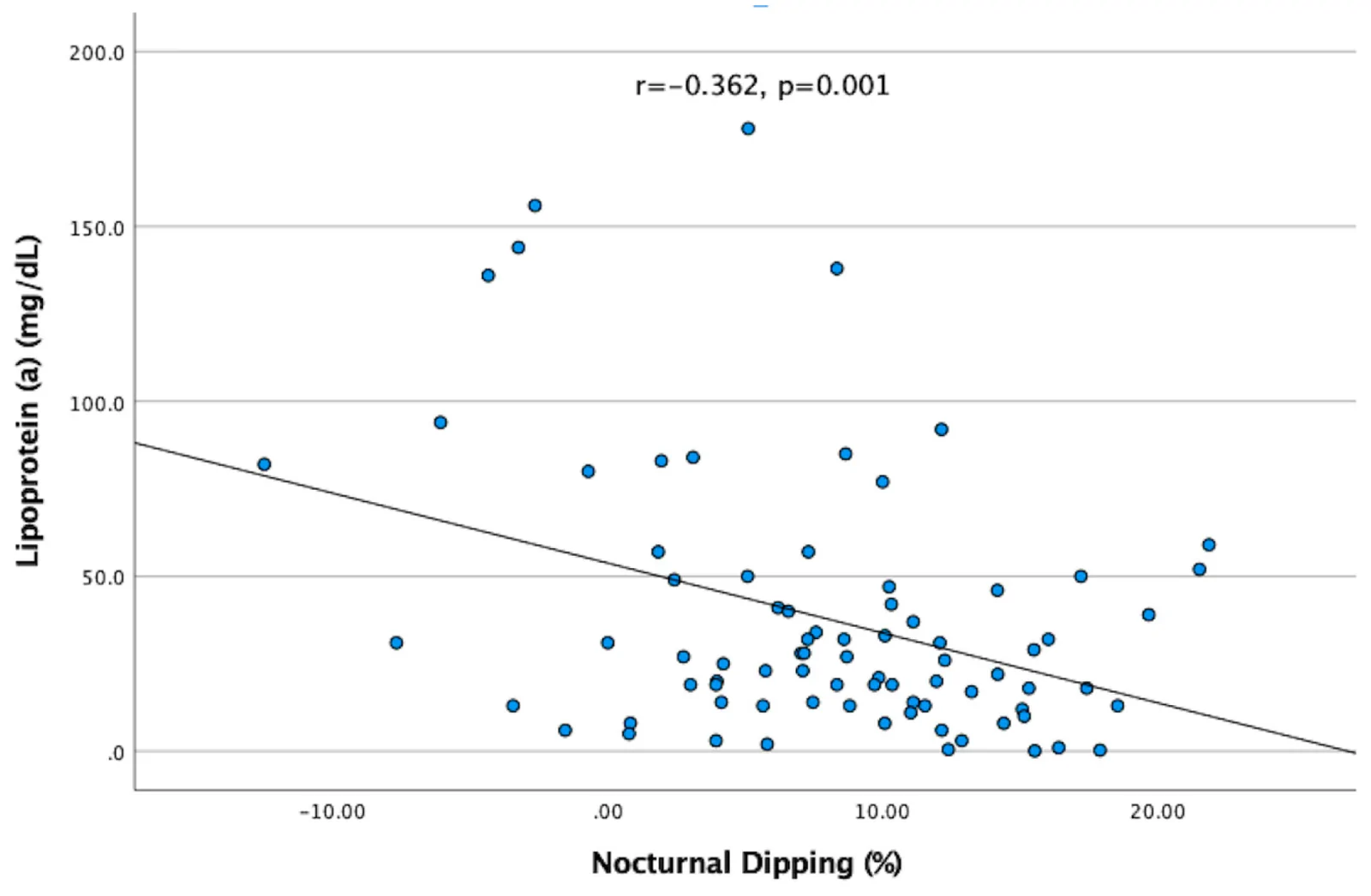
Correlation between serum Lp(a) concentration and nocturnal systolic blood pressure dipping percentage.

Serum Lp(a) concentration showed a significant inverse correlation with nocturnal systolic blood pressure dipping percentage (r = −0.362, *p* = 0.001) (Figure 1).

Scatter plot showing the relationship between serum lipoprotein(a) [Lp(a)] concentration (mg/dL) and nocturnal systolic blood pressure dipping percentage (%) in the study population. Pearson correlation analysis revealed a significant inverse correlation between Lp(a) levels and nocturnal dipping percentage (r = −0.362, *p* = 0.001), indicating that higher Lp(a) levels were associated with a reduced nocturnal blood pressure decline.

### 3.4. Multivariable Regression Analyses

In multivariable logistic regression analysis adjusted for age, sex, LDL cholesterol, and active smoking, elevated Lp(a) levels remained independently associated with carotid plaque presence (OR 3.24, 95% CI 1.06–9.80, *p* = 0.039) (Table 3). None of the other covariates showed an independent association with carotid plaque.

When analyzed as a continuous variable, higher serum Lp(a) concentrations were significantly associated with carotid plaque presence in univariable logistic regression analysis (OR 1.014 per 1 mg/dL increase, 95% CI 1.001–1.027, *p* = 0.036).

In multiple linear regression analysis adjusted for age, sex, LDL cholesterol, and office systolic blood pressure, higher Lp(a) levels remained independently associated with lower nocturnal systolic blood pressure dipping percentage (β = −0.294, *p* = 0.008). Increasing age was also independently associated with reduced dipping percentage (β = −0.271, *p* = 0.023), whereas male sex was positively associated with nocturnal dipping (β = 0.233, *p* = 0.029). LDL cholesterol and office systolic blood pressure were not independently associated with nocturnal dipping percentage (Table 4).

## 4. Discussion

The present study demonstrated that elevated serum Lp(a) levels were independently associated with carotid plaque presence and impaired nocturnal blood pressure decline in patients with hypertension. In contrast, no significant associations were observed between Lp(a) levels and carotid IMT, LVH, LVMI, hypertensive retinopathy, or renal functional parameters. Additionally, serum Lp(a) concentrations were inversely correlated with nocturnal dipping percentage and remained significantly associated with reduced dipping percentage after adjustment for age, sex, LDL cholesterol, and office systolic blood pressure. This finding suggests that the relationship between Lp(a) and impaired nocturnal blood pressure decline may not be explained solely by hypertension severity.

One of the principal findings of the present study was the independent association between elevated Lp(a) levels and carotid plaque presence, even after adjustment for conventional vascular risk factors including age, sex, LDL cholesterol, and active smoking. Lp(a) is increasingly recognized as a genetically determined cardiovascular risk factor with proatherogenic, proinflammatory, and prothrombotic properties [3,4]. Mechanistically, Lp(a) promotes endothelial dysfunction, oxidative stress, vascular inflammation, smooth muscle cell proliferation, and foam cell formation through oxidized phospholipid-mediated pathways [5,19,20]. These mechanisms contribute predominantly to focal plaque formation rather than diffuse arterial remodeling. Previous studies similarly demonstrated stronger associations between Lp(a) and carotid plaque burden than between Lp(a) and carotid IMT [11,12]. Moreover, this association remained significant when Lp(a) was analyzed as a continuous variable rather than only according to the predefined 50 mg/dL threshold, suggesting that the observed relationship was not solely dependent on categorical classification. Our findings are therefore consistent with the concept that carotid plaque assessment may better reflect Lp(a)-related vascular injury than IMT measurements. Furthermore, elevated Lp(a) has also been associated with recurrent cardiovascular events in patients with premature coronary artery disease, supporting its broader role in atherosclerotic cardiovascular risk. However, unlike that longitudinal prognostic study, our cross-sectional investigation evaluated associations with subclinical hypertension-mediated organ damage rather than clinical cardiovascular outcomes [21].

Importantly, no significant association was observed between Lp(a) and carotid IMT. This distinction is pathophysiologically relevant because carotid IMT is thought to reflect diffuse medial hypertrophy and chronic hemodynamic adaptation, whereas carotid plaque represents a more advanced and inflammation-driven atherosclerotic phenotype [9,10]. The absence of a significant relationship between Lp(a) and carotid IMT in our cohort further supports the hypothesis that Lp(a)-mediated vascular injury is more strongly linked to focal atherosclerotic processes than to generalized arterial wall thickening.

Another major finding of this study was the association between elevated Lp(a) levels and impaired nocturnal blood pressure decline. Serum Lp(a) concentrations were inversely correlated with nocturnal dipping percentage and remained independently associated with reduced dipping percentage after multivariable adjustment. Furthermore, higher Lp(a) levels were independently associated with lower nocturnal dipping percentage in linear regression analysis.

Although the precise mechanisms underlying this association remain uncertain, endothelial dysfunction, impaired nitric oxide bioavailability, arterial stiffness, oxidative stress, vascular inflammation, and autonomic dysregulation may contribute to impaired nocturnal blood pressure decline [13,14,20,22]. Since Lp(a) contributes to vascular inflammation and endothelial injury, elevated Lp(a) concentrations may identify hypertensive patients with a more adverse vascular phenotype and abnormal circadian blood pressure regulation. Recent observational data also demonstrated associations between elevated Lp(a) concentrations and ambulatory blood pressure abnormalities beyond conventional office blood pressure measurements, supporting the potential relationship between Lp(a) and altered blood pressure variability [23]. Age-related arterial stiffening and autonomic dysfunction may additionally contribute to impaired nocturnal blood pressure decline in hypertensive patients.

Despite these findings, no significant associations were identified between Lp(a) and LVH, LVMI, hypertensive retinopathy, or renal functional parameters. Previous studies investigating relationships between Lp(a) and hypertension-mediated organ damage have produced inconsistent results [19,24]. While some reports demonstrated associations between elevated Lp(a) concentrations and cardiac structural abnormalities or renal dysfunction, others failed to confirm these relationships after adjustment for conventional cardiovascular risk factors [19]. The lack of association between Lp(a) and echocardiographic markers of cardiac remodeling in our study may reflect the distinct pathophysiological mechanisms underlying vascular atherosclerosis and myocardial remodeling. Whereas Lp(a) primarily promotes focal atherosclerotic plaque formation through proatherogenic and proinflammatory pathways, left ventricular hypertrophy is predominantly driven by chronic pressure overload, arterial stiffness, neurohormonal activation, and the cumulative duration of hypertension. Moreover, the high prevalence of left ventricular hypertrophy observed in both study groups suggests that long-standing hypertension itself may have been the dominant determinant of myocardial remodeling, potentially masking any additional contribution of Lp(a). Finally, the relatively modest sample size may also have limited our ability to detect weaker associations between Lp(a) and cardiac structural parameters.

The relatively high left ventricular ejection fraction observed in both study groups should also be interpreted in the context of the study population. All participants had preserved systolic function, and patients with coronary artery disease, previous myocardial infarction, heart failure, or significant valvular heart disease were excluded according to the predefined study criteria. Therefore, the cohort primarily consisted of ambulatory hypertensive patients without overt structural heart disease. In this setting, preserved or even supranormal ejection fraction may reflect compensatory ventricular function during the early stages of pressure-overload remodeling rather than enhanced myocardial contractility. Although supranormal ejection fraction has been associated with increased mortality in large observational cohorts, these findings were derived from heterogeneous populations with established cardiovascular disease and should not be directly extrapolated to our relatively low-risk hypertensive cohort [25]. Accordingly, the high mean ejection fraction observed in our study is more likely attributable to the characteristics of the study population than to serum Lp(a) concentrations. Nevertheless, the prognostic significance of supranormal ejection fraction could not be evaluated in this cross-sectional study and warrants further investigation.

Similarly, we did not observe a significant association between serum Lp(a) levels and hypertensive retinopathy. Previous studies evaluating the relationship between Lp(a) and retinal disease have predominantly focused on diabetic retinopathy and generally reported higher Lp(a) concentrations among patients with diabetic retinal microvascular complications. Recent systematic reviews and a meta-analysis have likewise concluded that elevated Lp(a) levels are associated with both the presence and severity of diabetic retinopathy [26,27]. However, unlike those studies, our cohort exclusively included nondiabetic patients with essential hypertension. Therefore, the absence of an association in our study may reflect differences in the underlying pathophysiology of hypertensive and diabetic retinal disease, as well as the exclusion of diabetes mellitus and chronic kidney disease, both of which are known to influence retinal microvascular injury. Accordingly, our findings suggest that elevated Lp(a) may show a stronger association with carotid plaque than with hypertensive retinal abnormalities in this carefully selected nondiabetic hypertensive cohort; however, this observation should be interpreted cautiously because residual confounding and the cross-sectional study design preclude causal inference.

Several limitations should be acknowledged. First, the retrospective cross-sectional design precludes causal inference. Second, the relatively small sample size and limited number of patients with elevated Lp(a) levels may have reduced statistical power, particularly for secondary outcomes, and contributed to imprecise effect estimates. In particular, the relatively wide confidence interval observed for the association between elevated Lp(a) levels and carotid plaque presence indicates limited precision regarding the magnitude of this association. Therefore, although the association remained statistically significant, it should be interpreted cautiously until confirmed in larger, adequately powered prospective studies. Third, the study was conducted at a single center, which may limit the generalizability of our findings. Furthermore, ethnicity-specific variation in Lp(a) concentrations may influence the observed associations; however, ethnic background was not systematically recorded in our cohort [28]. Fourth, longitudinal cardiovascular outcome data were unavailable; therefore, the prognostic significance of the observed associations could not be assessed. Fifth, inflammatory biomarkers and direct measures of arterial stiffness were not systematically assessed. In addition, several potentially important determinants of carotid atherosclerosis and nocturnal blood pressure dipping, including hypertension duration, long-term blood pressure control, timing of antihypertensive medication administration, statin use, menopausal status, physical activity, alcohol consumption, sleep quality, and obstructive sleep apnea, were not consistently available because of the retrospective study design. Therefore, residual confounding cannot be excluded despite multivariable adjustment. Moreover, carotid atherosclerosis was evaluated using plaque presence as a binary variable. Quantitative data regarding plaque burden, plaque morphology, plaque score, and inter- or intra-observer reproducibility were not routinely recorded in our retrospective database and were therefore unavailable for analysis. Finally, although ambulatory blood pressure monitoring parameters were comprehensively evaluated, repeated ABPM recordings were not available, which may have influenced the assessment of circadian blood pressure reproducibility.

## 5. Conclusions

Elevated serum Lp(a) levels were independently associated with carotid plaque presence and impaired nocturnal blood pressure decline in patients with hypertension. In contrast, no significant associations were observed between Lp(a) and carotid IMT, LVH, LVMI, hypertensive retinopathy, or renal functional parameters.

These findings suggest that elevated Lp(a) may be associated with a less favorable vascular profile characterized by carotid plaque and impaired nocturnal blood pressure regulation in patients with hypertension. However, given the retrospective cross-sectional design and the relatively small sample size, these findings should be interpreted with caution and confirmed in larger prospective studies.

## Figures and Tables

**Table 1:** Baseline demographic, biochemical, echocardiographic, and vascular characteristics of the study population according to lipoprotein(a) levels.

Variable	Lp(a) < 50 mg/dL (*n* = 60)	Lp(a) ≥ 50 mg/dL (*n* = 20)	*p* Value
Age, years	53.1 ± 6.9	57.6 ± 9.3	0.024
Male sex, *n* (%)	20 (33.3)	7 (35.0)	0.891
BMI, kg/m^2^	28.1 ± 3.2	27.0 ± 3.1	0.182
Heart rate, bpm	73.8 ± 11.3	74.5 ± 12.1	0.814
Office SBP, mmHg	150.3 ± 16.0	157.8 ± 13.8	0.064
Office DBP, mmHg	91.6 ± 7.9	92.3 ± 8.7	0.750
Lp(a), mg/dL	20.1 ± 12.4	90.1 ± 39.3	<0.001
Total cholesterol, mg/dL	218.1 ± 36.8	224.2 ± 34.6	0.514
LDL cholesterol, mg/dL	138.2 ± 35.3	137.7 ± 30.5	0.956
HDL cholesterol, mg/dL	51.2 ± 14.9	56.5 ± 16.1	0.182
Triglycerides, mg/dL	118 (90.8–175)	111.5 (94.3–170)	0.929
eGFR, mL/min/1.73 m^2^	101.7 ± 26	96.1 ± 30.1	0.436
LVMI, g/m^2^	120.3 ± 25.1	129.7 ± 34.8	0.193
Mean carotid IMT, mm	0.78 ± 0.13	0.76 ± 0.12	0.607
Maximum carotid IMT, mm	0.97 ± 0.17	0.95 ± 0.14	0.534
Carotid cross-sectional area, mm^2^	16.3 ± 3.9	16.4 ± 3.7	0.904
Ejection fraction, %	72.9 ± 5.6	75.4 ± 5.3	0.089
IVS thickness, mm	11.3 ± 1.8	11.9 ± 2.2	0.211
PW thickness, mm	10.6 ± 1.4	10.7 ± 1.7	0.860
Active smoking, *n* (%)	10 (16.7)	6 (30.0)	0.197
Beta-blocker use, *n* (%)	12 (20)	4 (20)	1.000
ACE inhibitor use, *n* (%)	20 (33.3)	7 (35.0)	0.891
ARB use, *n* (%)	10 (16.7)	5 (25.0)	0.408

Data are presented as mean ± standard deviation, median (interquartile range), or number (percentage), as appropriate. Comparisons between groups were performed using the independent samples *t*-test, Mann–Whitney U test, or chi-square test, according to data distribution.

**Table 2:** Comparison of hypertension-mediated organ damage markers and circadian blood pressure patterns according to lipoprotein(a) levels.

Variable	Lp(a) < 50 mg/dL (*n* = 60)	Lp(a) ≥ 50 mg/dL (*n* = 20)	*p* Value
Carotid plaque, *n* (%)	14 (23.3)	11 (55.0)	0.008
LVH on echocardiography, *n* (%)	32 (53.3)	13 (65.0)	0.362
Hypertensive retinopathy, *n* (%)	50 (83.3)	16 (80)	0.485
Non-dipper pattern, *n* (%)	28 (46.7)	15 (75.0)	0.028
Nocturnal dipping percentage (%)	9.96 (5.75–13.15)	4.09 (−2.16–9.66)	0.015
24 h urinary protein, mg/day	120.75 (90.4–173.7)	120.60 (89.9–202.8)	0.907
24 h urinary albumin, mg/day	6.16 (2.57–10.15)	5.10 (2.42–9.14)	0.965

Data are presented as number (percentage) or median (interquartile range), as appropriate. Group comparisons were performed using the chi-square test or Mann–Whitney U test. Lp(a), lipoprotein(a); LVH, left ventricular hypertrophy.

**Table 3:** Multivariable logistic regression analysis for carotid plaque presence.

Variable	OR	95% CI	*p* Value
Age	1.047	0.976–1.124	0.197
Male sex	1.131	0.362–3.536	0.832
LDL cholesterol	1.006	0.990–1.022	0.458
Active smoking	0.597	0.172–2.075	0.417
Elevated Lp(a)	3.24	1.06–9.80	0.039

Variables entered into the multivariable logistic regression model were age, sex, LDL cholesterol, active smoking, and elevated Lp(a). Elevated Lp(a) was defined as serum Lp(a) ≥ 50 mg/dL. Odds ratios are presented with 95% confidence intervals. Model calibration was assessed using the Hosmer–Lemeshow goodness-of-fit test. Multivariable logistic regression analysis was performed using the enter method. Model fit statistics: Cox–Snell R^2^ = 0.110; Nagelkerke R^2^ = 0.155. OR, odds ratio; CI, confidence interval; Lp(a), lipoprotein(a); LDL, low-density lipoprotein.

**Table 4:** Multiple linear regression analysis for determinants of nocturnal blood pressure dipping percentage.

Variable	Standardized β	95% CI for B	*p* Value
Age	−0.271	−0.443 to −0.033	0.023
Male sex	0.233	0.345 to 6.345	0.029
Lp(a)	−0.294	−0.092 to −0.014	0.008
LDL cholesterol	−0.083	−0.058 to 0.025	0.426
Office SBP	0.148	−0.030 to 0.158	0.180

Standardized beta coefficients (β), unstandardized coefficient confidence intervals, and *p* values are presented. Variables entered into the multiple linear regression model were age, sex, Lp(a), LDL cholesterol, and office systolic blood pressure (SBP). β, standardized beta coefficient; CI, confidence interval; Lp(a), lipoprotein(a); LDL, low-density lipoprotein; SBP, systolic blood pressure.

## Data Availability

The data presented in this study are available from the corresponding author upon reasonable request. The data are not publicly available because they contain information that could compromise participant privacy and are subject to ethical restrictions.

## Abbreviations

The following abbreviations are used in this manuscript:

ABPM	Ambulatory Blood Pressure Monitoring
ACE	Angiotensin-Converting Enzyme
ARB	Angiotensin Receptor Blocker
BMI	Body Mass Index
CI	Confidence Interval
DBP	Diastolic Blood Pressure
eGFR	Estimated Glomerular Filtration Rate
HDL	High-Density Lipoprotein
IMT	Intima–Media Thickness
IVS	Interventricular Septum
LDL	Low-Density Lipoprotein
Lp(a)	Lipoprotein(a)
LVH	Left Ventricular Hypertrophy
LVMI	Left Ventricular Mass Index
OR	Odds Ratio
PW	Posterior Wall
SBP	Systolic Blood Pressure
